# The Origin, Epidemiology, and Phylodynamics of Human Immunodeficiency Virus Type 1 CRF47_BF

**DOI:** 10.3389/fmicb.2022.863123

**Published:** 2022-05-16

**Authors:** Gracelyn Hill, Marcos Pérez-Losada, Elena Delgado, Sonia Benito, Vanessa Montero, Horacio Gil, Mónica Sánchez, Javier E. Cañada-García, Elena García-Bodas, Keith A. Crandall, Michael M. Thomson, the Spanish Group for the Study of New HIV Diagnoses, José Luis Díaz de Tuesta del Arco

**Affiliations:** Hospital Universitario de Basurto; Hospital Universitario de Cruces, Bilbao; Hospital Universitario de Galdakao; Hospital Universitario Donostia, San Sebastián; Hospital Universitario Araba, Vitoria; Hospital Universitario de Navarra, Pamplona; Hospital Reina Sofía, Tudela; Hospital Universitario Sant Joan d’Alacant; Complejo Hospitalario Universitario de Vigo; Complejo Hospitalario Universitario de Pontevedra; Complejo Hospitalario Lucus Augusti, Lugo; Complejo Hospitalario Universitario de Ourense; Hospital Universitario Miguel Servet, Zaragoza; Centro Sanitario Sandoval, Madrid; Hospital Universitario Río Hortega, Valladolid; Hospital Universitario de Toledo; ^1^Computational Biology Institute, George Washington University, Washington, DC, United States; ^2^Department of Biostatistics and Bioinformatics, Milken Institute School of Public Health, George Washington University, Washington, DC, United States; ^3^CIBIO-InBIO, Centro de Investigação em Biodiversidade e Recursos Genéticos, Universidade do Porto, Porto, Portugal; ^4^HIV Biology and Variability Unit, Centro Nacional de Microbiología, Instituto de Salud Carlos III, Madrid, Spain

**Keywords:** HIV, phylodynamics, circulating recombinant form (CRF), Spain, epidemiology, CRF47_BF

## Abstract

CRF47_BF is a circulating recombinant form (CRF) of the human immunodeficiency virus type 1 (HIV-1), the etiological agent of AIDS. CRF47_BF represents one of 19 CRFx_BFs and has a geographic focus in Spain, where it was first identified in 2010. Since its discovery, CRF47_BF has expanded considerably in Spain, predominantly through heterosexual contact (∼56% of the infections). Little is known, however, about the origin and diversity of this CRF or its epidemiological correlates, as very few samples have been available so far. This study conducts a phylogenetic analysis with representatives of all CRFx_BF sequence types along with HIV-1 M Group subtypes to validate that the CRF47_BF sequences share a unique evolutionary history. The CRFx_BF sequences cluster into a single, not well supported, clade that includes their dominant parent subtypes (B and F). This clade also includes subtype D and excludes sub-subtype F2. However, the CRF47_BF sequences all share a most recent common ancestor. Further analysis of this clade couples CRF47_BF protease-reverse transcriptase sequences and epidemiological data from an additional 87 samples collected throughout Spain, as well as additional CRF47_BF database sequences from Brazil and Spain to investigate the origin and phylodynamics of CRF47_BF. The Spanish region with the highest proportion of CRF47_BF samples in the data set was the Basque Country (43.7%) with Navarre next highest at 19.5%. We include in our analysis epidemiological data on host sex, mode of transmission, time of collection, and geographic region. The phylodynamic analysis indicates that CRF47_BF originated in Brazil around 1999–2000 and spread to Spain from Brazil in 2002–2003. The virus spread rapidly throughout Spain with an increase in population size from 2011 to 2015 and leveling off more recently. Three strongly supported clusters associated with Spanish regions (Basque Country, Navarre, and Aragon), together comprising 60.8% of the Spanish samples, were identified, one of which was also associated with transmission among men who have sex with men. The expansion in Spain of CRF47_BF, together with that of other CRFs and subtype variants of South American origin, previously reported, reflects the increasing relationship between the South American and European HIV-1 epidemics.

## Introduction

High genetic diversity of human immunodeficiency virus type 1 (HIV-1) is a defining feature of the AIDS virus. This diversity gain and loss is a hallmark of the evolution of HIV in the context of drug resistance and changing environments (Pennings et al., 2014). A contributing factor in the evolution of HIV is the process of recombination (Rambaut et al., 2004; Vuilleumier and Bonhoeffer, 2015). Genetic recombination is known to impact HIV allelic diversity and subsequent population dynamics at a rate equivalent to the high mutation rate of HIV (Shriner et al., 2004). Genetic diversity within HIV subtypes can be up to 17% sequence divergence across the genome with 17–35% divergence between subtypes (Castro-Nallar et al., 2012a). Yet recombination can even occur between subtypes as HIV variants spread around the globe, leading to circulating recombinant forms or CRFs, as well as unique recombinant forms (URFs) (Castro-Nallar et al., 2012b). There are currently 118 known HIV-1 CRFs according to the Los Alamos HIV Sequence Database (Los Alamos National Laboratory, 2021) involving recombination events between nearly all known subtypes and even between other CRFs [e.g., CRF15_01B is a recombinant form between CRF01 and subtype B (Tovanabutra et al., 2003)]. The CRFs often have their own unique population dynamics and molecular epidemiology compared to their parental strains and often lead to novel infection dynamics and spread. One such CRF is CRF47_BF, discovered in Spain and described in 2010 (Fernández-García et al., 2010) as an intersubtype recombinant form between HIV-1 subtypes B and F. Of the CRFs, among the most abundant are those between B and F subtypes, with 19 CRF_BFs (note that in the Los Alamos HIV Database these are sometimes designated “BF” and sometimes “BF1,” even for the same CRF). Of the CRF_BFs, all but two are known from South America (mainly Brazil, but Argentina, Uruguay, Paraguay, Chile, Peru, and Bolivia as well) with a few found both in South America and Europe (CRF66, 75, and 89). Only two CRF_BF have been reported to be found circulating exclusively in Europe, CRF42_BF in Luxembourg (Struck et al., 2015) and CRF47_BF in Spain (Fernández-García et al., 2010). Since its description, CRF47_BF has expanded considerably in Spain, predominantly *via* heterosexual contact, and is now known from Brazil as well, as attested by a CRF47_BF virus collected in this country whose sequence is deposited in the Los Alamos database (Los Alamos National Laboratory, 2021).

The goal of this study is to estimate the temporal and geographic origin of CRF47_BF and the dynamics of diffusion and growth throughout its evolutionary history. Toward this goal, we combine new CRF47_BF sequence data from our lab from strains isolated in Spain with data from other BF strains in the Los Alamos database to examine the origin and evolutionary dynamics of CRF47_BF and their epidemiological correlates.

## Materials and Methods

### Sample and Data Collection

Plasma and whole blood samples were collected from HIV-1-infected patients at public hospitals across eight regions in Spain for a molecular epidemiological study of all new HIV-1 diagnoses seen at the participating centers and for antiretroviral drug resistance testing. Epidemiological data from the CRF47_BF patients were collected to link to the HIV sequence data. The epidemiological data included patient gender, the transmission route, the patient’s year of HIV diagnosis and date of sample collection, the region from which the sample was collected, the country of origin of the individual, and whether the patient was on antiretroviral (ARV) therapy.

The study was approved by the Committee of Research Ethics of Instituto de Salud Carlos III, Majadahonda, Madrid, Spain (report numbers CEI PI 38_2016-v3 and CEI PI 31_2019-v5). The study did not require written informed consent by the study participants, as it used samples and data collected as part of routine clinical practice and patients’ data were anonymized without retaining data allowing individual identification.

### Sequence Analyses

(RT-)PCR was used to amplify the protease-reverse transcriptase (PR-RT) gene region from plasma-extracted RNA or whole blood-extracted DNA using previously described primers (Delgado et al., 2015; Supplementary Figure 1). PCR products were sequenced using the Sanger method with an automated capillary sequencer. These data were combined with PR-RT sequences classified as CRF47_BF at the Los Alamos HIV Sequence Database and reference sequences for all subtypes and all CRFx_BFs for this same gene region from the Los Alamos HIV Database. Finally, we conducted a BLAST (Altschul et al., 1990) search against GenBank with the 5′-most 950 nt of PR-RT of all CRF47_BF viruses and included all sequences within 95% similarity. BLAST searches and further analyses (see below) yielded only two additional CRF47_BF sequences not identified as such at the Los Alamos database (with GenBank accessions JF929086, from Spain, and JQ238096, from Brazil).

We conducted two analyses with these data. (1) We included all data to validate the quality of the data and place the CRF47_BF within a broader phylogenetic context. Our initial phylogenetic analysis included subtypes from the HIV-1 M group (subtypes A1, A2, B, C, D, F1, F2, G, H, J, K, and L), as well as the CRF_BF recombinants (TotalCRF_BF.fasta, see Supplementary Material). Our final alignment (1,200 bp) included 14 sequences representing all the major subtypes within HIV-1 group M, 5 subtype B sequences, 11 subtype F (F1, F2) sequences, and 34 representatives of all known and distinct CRF_BFs. This alignment also included our more focused (2) CRF47_BF dataset (CRF47_BF.fasta see Supplementary Material). By including additional subtypes (including lab strains), we can both verify the monophyly of our target group of CRF47_BF sequences and validate that there are not contaminants or strange recombinants within this group as would be indicated by novel phylogenetic placement. For this second dataset, we included all 99 sequences from CRF47_BF, including 87 obtained by us [7 from a previous study (Fernández-García et al., 2010) and 80 newly derived] from the patients summarized in Table 1, and 12 from databases (10 from Spain and 2 from Brazil). We then conducted a focused analysis on the targeted CRF47_BF strains (1,377 aligned bp).

**TABLE 1:** Summary data from patients with CRF47_BF variant from Spain.

	Total*N* = 87	Percent	New sequences
**Gender**			
Male	68	78.2	
Female	18	20.7	
Transgender	1	1.2	
**Region**			
Basque Country	38	43.7	35 (38 total)
Navarre	17	19.5	17
Galicia	14	16.1	10 (14 total)
Aragon	9	10.3	9
Comunitat Valenciana	6	6.9	6 (13 total)
Castilla y Leon	1	1.2	1
Castilla-La Mancha	1	1.2	1
Madrid	1	1.2	1
**Transmission route**			
Heterosexual	49	56.3	
MSM	19	21.8	
Sexual transmission (unspecified sexuality)	12	13.8	
Other/no data	7	8.1	
**ARV therapy**			
No	75	86.2	
Yes	7	8.1	
No data	5	5.7	
**Country of origin**			
Spain	68	78.2	
Brazil	5	5.8	
Colombia	5	5.8	
Morocco	3	3.5	
Nicaragua	2	2.3	
Other	4	4.6	

In both analyses, we aligned sequence data using MAFFT (Katoh and Standley, 2013) with the FFT-NS-2 progressive alignment approach since these sequences are relatively similar. Prior to subsequent phylogenetic and phylodynamic analyses, we checked that all sequences showed mosaic structures coincident with CRF47_BF, through two procedures: (1) bootscan analyses and (2) separate phylogenetic trees of B and F1 segments previously defined for CRF47_BF (Fernández-García et al., 2010), including B and F1 subtype references, to ensure that subtype assignment of each segment was identical for all sequences. Phylogenetic analyses were conducted using maximum-likelihood (Felsenstein, 1981; Posada and Crandall, 2021) as implemented by RAxML (Kozlov et al., 2019) *via* the CIPRES web service (Miller et al., 2012). The phylogenetic analyses utilized the best-fit model of evolution (Posada and Crandall, 1998) as determined by ModelTest-NG (Darriba et al., 2020). Phylogenetic analyses were also done using a Bayesian approach as implemented by MrBayes 3.2 (Ronquist et al., 2012) with integrated model selection, 10 million MCMC generations, and codon partitioning. Confidence in the resulting phylogenetic estimates was assessed using the bootstrap approach (Felsenstein, 1985) for the maximum-likelihood analyses with 1,000 pseudoreplicates and with posterior probabilities (pP) in the Bayesian framework. Phylogenetic trees were visualized with iTOL (Letunic and Bork, 2019), as well as mapping of epidemiological characters along the phylogeny.

We applied BEAST2 (Bouckaert et al., 2014) to the CRF47_BF dataset to estimate a chronogram and the phylodynamic history of CRF47_BF. First, we validated the existence of temporal signal in the dataset with TempEst v1.5.3 (Rambaut et al., 2016), which determines the correlation of genetic divergence among sequences (measured as root-to-tip distance) with time. For the BEAST2 analysis we ran 10 million generations, two codon partitions (1st + 2nd, and 3rd positions), used an uncorrected log-normal relaxed molecular clock (initial ucld.mean = 1.0 and initial ucld.stdev = 0.333), estimated base frequencies and the HKY + G evolution model. The input file was created using BEAUti. Past population dynamics was estimated *via* Skygrid analysis (Hill and Baele, 2019) using a coalescent Bayesian Skygrid tree prior. We used Tracer (Rambaut et al., 2018) to verify convergence and to visualize the Skygrid plot. We compare the inferred effective population size of the CRF47_BF population in Spain to the proportion of CRF47_BF diagnoses over time across the same study regions and time period.

Finally, known drug resistant mutations were identified in the focused CRF47_BF data using the Stanford HIV Drug Resistance Database’s HIVdb v9.0 program (Tang et al., 2012).

### Statistical Analyses

Correlations between cluster membership and epidemiological data were analyzed with Fisher’s exact test.

## Results

### Epidemiology and Sequences

**FIGURE 1:**
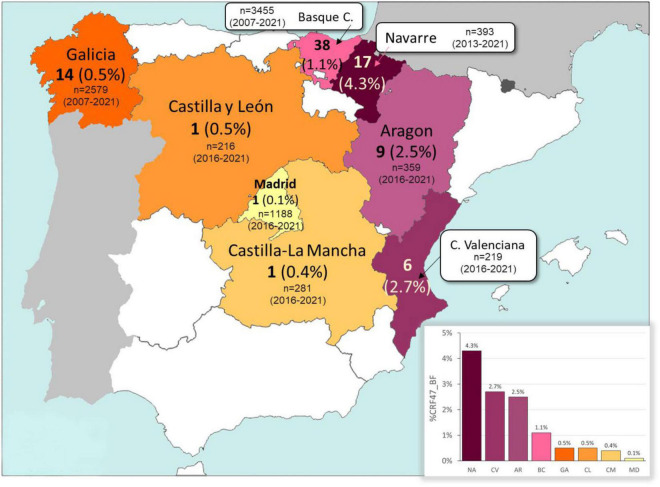
Map of Spain with the number and estimated prevalence (percentage between parentheses) of HIV-1 CRF47_BF viruses detected in each of the eight regions analyzed. The number of new HIV-1 diagnoses (*n*) and the period of study in each region are also indicated. The percentage of CRF47_BF in each region is also represented in the inserted bar chart. NA, Navarre; CV, Comunitat Valenciana; AR, Aragon; BC, Basque Country; GA, Galicia; CL, Castilla y León; CM, Castilla-La Mancha; MD, Madrid.

We collected samples and epidemiological data from 87 patients throughout eight different regions of Spain (Basque Country, Navarre, Galicia, Aragon, Comunitat Valenciana, Madrid, Castilla-La Mancha, and Castilla y León) (Figure 1). Collections were made from 2007 to 2021. Males accounted for 78% of the individuals with CRF47_BF in our study and 56% of individuals reported transmission *via* heterosexual contact (61% considering only individuals with available data on transmission route) (Table 1). The Spanish region with the highest proportion of the CRF47_BF variant in our data set was the Basque Country with 44% of the cases, while Navarre was the next highest (18% of the cases) (see Table 1 for number of new CRF47_BF sequences and total CRF47_BF sequences per region, and Figure 1 for the total number of analyzed HIV-1 sequences and prevalence of CRF47_BF among new HIV-1 diagnoses in each region in the sampling periods). Most samples were collected shortly after HIV diagnosis. Patients received ARV therapy after sample collection.

### Phylogenetics

The first phylogenetic analysis was a maximum likelihood phylogenetic estimate of the relationships amongst the CRFx_BFs, including HIV-1 M subtypes as outgroup taxa and subtypes B, F, and CRFx_BFs as ingroup taxa. Our RAxML tree depicted a monophyletic cluster of the subtype B, F, and CRF_BFs relative to the other HIV-1 subtypes (Supplementary Figure 2), but including also Subtype D. The backbone structure of the CRF phylogenetic relationships was weakly supported (<70% bootstrap support – indicated by dashed lines), which is not particularly surprising given the potential difficulty in representing evolutionary histories of recombinant HIV-1 forms as bifurcating trees (Posada and Crandall, 2001, 2002). Many of the CRFx_BF forms cluster in strongly supported monophyletic groups themselves (e.g., CRF40_BF, CRF72_BF, CRF75_BF, CRF90_BF, CRF89_BF, etc.), including our target group of CRF47_BF sequences. Many of the other CRFs form weakly supported monophyletic groups (e.g., CRF70_BF, CRF46_BF, CRF38_BF, etc.) and a few form non-monophyletic groupings (e.g., CRF66_BF and CRF71_BF). The subtype B sequences cluster together within the CRFx_BF clade with both a cluster of subtype D and the CRF28_BF sequence nested within this subtype B cluster. Nevertheless, the target group for this study, the CRF47_BF sequences, clearly form a monophyletic group, suggesting independent evolution, and are a sister group to the CRF44_BF clade.

**FIGURE 2:**
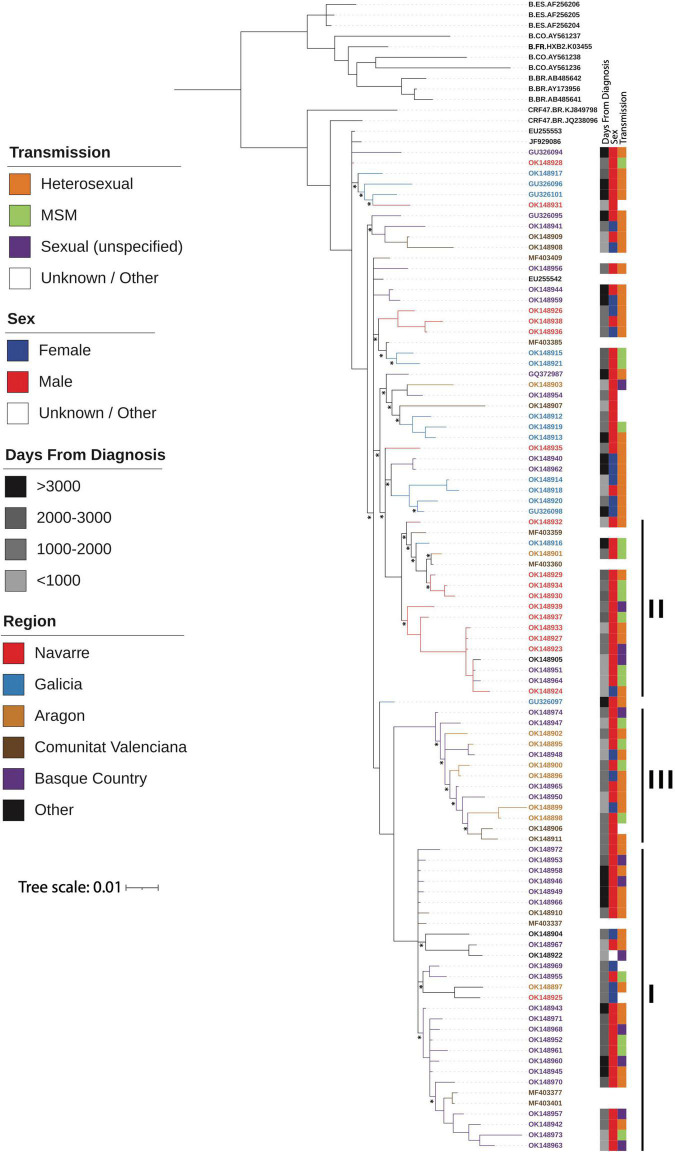
Majority-rule consensus Bayesian (MrBayes) phylogenetic estimate of CRF47_BF sequences from Spain (colored by region) and other CRF47_BF sequences from GenBank, as well as a few additional subtype B sequences from Spain, Brazil, and Colombia plus HXB2, all to serve as an outgroup to the CRF47 sequences. Only clade posterior probabilities <0.95 are indicated by an *; all other clades showed posterior probabilities ≥0.95. Epidemiological data are mapped to the right of the phylogeny, including days from diagnosis, sex, transmission, and geographic region. Branch lengths are drawn proportional to the amount of sequence divergence. Clusters corresponding to the Spanish regions of Basque Country (cluster I), Navarre (cluster II), and Aragon (cluster III) are indicated.

The Bayesian estimated phylogeny for the CRF47_BF sequences shows a monophyletic grouping of the sequences from Spain (Figure 2) with the two sequences from Brazil (KJ849798 and JQ238096) branching basally. Within the Spanish cluster, there are three strongly supported clusters, comprising 29 (cluster I), 17 (cluster II), and 13 (cluster III) viruses, respectively, which are associated with the Basque Country (*p* = 0.0002), Navarre (*p* = 0.0001), and Aragon (*p* = 0.0002), respectively. This is indicative of a single introduction of CRF47_BF into Spain with subsequent spread throughout the country and point introductions with subsequent expansion in different regions. The mixing of patient gender throughout the resulting phylogeny supports the epidemiological data suggesting predominantly heterosexual transmission among patients. We also found that cluster II, associated with Navarre, was associated with men who have sex with men (MSM) (*p* = 0.0388). In this cluster, 14 of 15 individuals with known gender are men.

**FIGURE 3:**
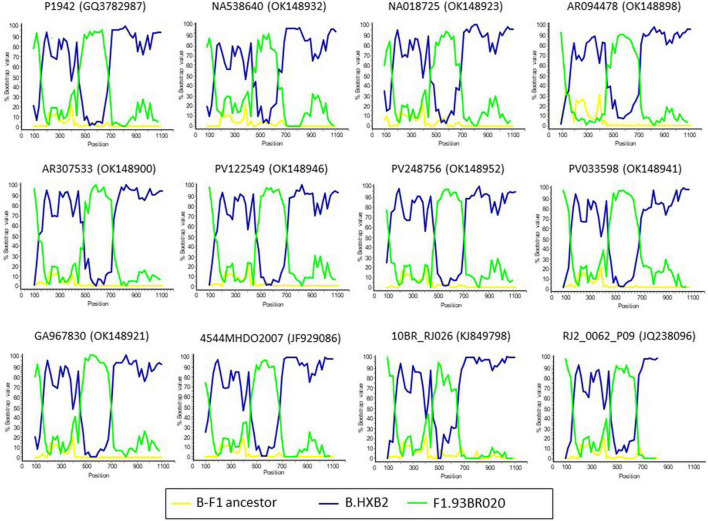
Bootscan analyses of PR-RT sequences of CRF47_BF viruses. Simplot v3.5 (Lole et al., 1999) was used for the analyses. Twelve representative profiles are displayed. Names of viruses, with GenBank accessions, are shown above each bootscan plot. P1942 was included as CRF47_BF reference. A reconstructed B-F1 ancestral sequence was used as outgroup. The horizontal axis represents the position from nucleotide 1 of protease and the vertical axis represents bootstrap values supporting clustering with references.

The bootscan analysis for recombination and separate trees of B and F segments suggest that all the target sequences within the CRF47_BF analyses presented here share the same recombination pattern (as outlined at the Los Alamos HIV Database) (Figure 3 and Supplementary Figure 3). Thus, while recombination can significantly impact phylogenetic interpretations (certainly, for the overall tree presented in Supplementary Figure 2), it does not seem to be differentially impacting analyses of our targeted CRF47_BF sequences.

Based on the sample dates, we grouped these in four temporal categories of recency (days between diagnosis date and current date) (>3,000, 2,000–3,000, 1,000–2,000, and <1,000 days from current) for ease of visualization of time over the phylogeny to test for temporal clustering. Thus, the greater the value the closer to the most recent common ancestor, i.e., origin of CRF47_BF. Note that these correspond well to the branch lengths observed leading to samples with <1,000 days from diagnosis having longer branches from the root to the terminal samples and >3,000 having shorter and more basal branches in the phylogram. No temporal clustering was observed as these different time categories were distributed throughout the CRF47_BF phylogeny (Figure 2).

### Analysis of Drug Resistance Mutations

To identify drug resistance mutations in the CRF47_BF viruses, we analyzed the sequences with the Stanford HIV Drug Resistance Database’s HIVdb program (Tang et al., 2012). We found ARV drug resistance mutations in five patients: M184V or M184I mutations of resistance to nucleoside reverse transcriptase inhibitors (NRTI) in three samples; K103N mutation of resistance to non-nucleoside reverse transcriptase inhibitors (NNRTI) + K65N mutation of resistance to NRTIs in one sample; and E138A mutation associated with low level resistance to the NNRTI rilpivirine in one patient. Only one of these patients, with M184I mutation, was ARV drug-experienced.

### Phylodynamics

**FIGURE 4:**
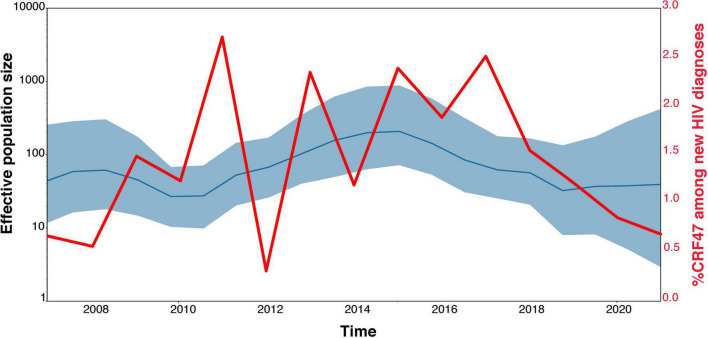
Population dynamics of CRF47_BF in Spain. Bayesian Skygrid estimate of fluctuating population size by year compared with the actual proportion of new CRF47_BF samples collected in the study each year among new HIV-1 diagnoses.

**FIGURE 5:**
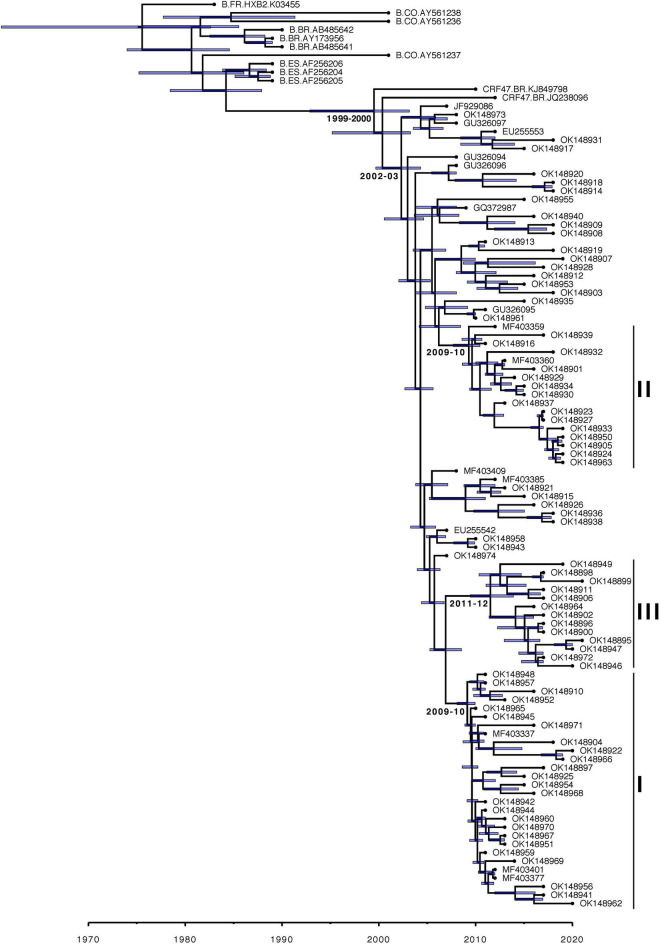
Maximum clade credibility Bayesian (BEAST) chronogram estimate of CRF47_BF sequences from Spain and other CRF47_BF sequences from GenBank, as well as a few additional subtype B sequences from Spain, Brazil, and Colombia plus HXB2, all to serve as an outgroup to the CRF47_BF sequences. Clusters associated with the Spanish regions of Basque Country (cluster I), Navarre (cluster II), and Aragon (cluster III) are indicated. Estimated years of emergence of CRF47_BF in Brazil, of its introduction in Spain, and of emergence of the Spanish clusters are indicated besides the corresponding nodes. A total of 95% highest posterior density (HPD) intervals (blue bars) are shown for all time estimates.

Our TempEst analysis determined that there was an adequate temporal signal in the dataset (*R*^2^ = 0.5051). With time-stamped sequence data, we performed a Bayesian Skygrid coalescent analysis to estimate historical population dynamics (Hill and Baele, 2019) of the CRF47_BF variants throughout Spain. Time labels (tipdates) were determined by the date of sample collection (ranging from 2007 to 2021). Our analysis supports a fairly dynamic population history of the CRF47_BF in Spain over the last 15 years with an initial increase in population size, a subsequent increase from 2011 to 2015, with a leveling off more recently, but seemingly increasing variance (Figure 4). This fluctuation in effective population size of CRF47_BF in Spain is not as dynamic as the % CRF47_BF infections among new HIV diagnoses, that fluctuate considerably over this same time period (Figure 4), but with similar overall trends. The average effective population size was estimated to be 155 with a mean substitution rate of 1.8128 × 10^−3^ [95% highest posterior density (HPD) interval (1.3956 × 10^–3^, 2.2548 × 10^–3^)]. Using BEAST, we estimated a chronogram to determine the time of origin for both the CRF47_BF clade as well as the timing of the introduction of CRF47_BF viruses to Spain (Figure 5). We estimated the origin of the CRF47_BF clade in Brazil (pP = 1.0), dated to 1999–2000 (95% HPD interval between 1994 and 2003) and timed the introduction of CRF47_BF to Spain (pP = 0.99) to be 2002–2003 (95% HPD interval between 2000 and 2004) (Figure 5). Similarly, viral strains seem to have entered once and spread through the Spanish regions of Basque Country (cluster I) (pP = 1.0), Navarre (cluster II) (pP = 1.0), and Aragon (cluster III) (pP = 1.0) between 2009 and 2012 (Figure 5). These analyses, hence, suggest that CRF47_BF was probably circulating in Spain for about 8 years before it was identified through DNA sequencing, but clearly at a relatively low frequency. Given the sampling of CRF47 sequences, it appears that the introduction of this recombinant form to Spain was *via* Brazil, supported by very high posterior probabilities (pP = 1.00).

## Discussion

The HIV-1 CRF47_BF was first reported in 2010, detected in nine samples collected in Spain in 2007–2009. Samples have subsequently been collected as this novel variant has spread throughout the country. Our phylogenetic analysis shows that isolates of CRF47_BF form a strongly supported monophyletic group (share a most recent common ancestor) relative to other CRFx_BF sequences, distinct from other CRFx_BF sequences, subtype B, sub-subtype F2 and other Group M subtypes. A focused phylogenetic analysis of the CRF47_BF sequences show a clear single origin in Brazil around 1999–2000 with a subsequent transmission and rapid spread throughout Spain beginning around 2002–2003. Three strongly supported clusters, comprising a majority of viruses and associated with the regions of Basque Country, Navarre, and Aragon, were identified; this suggests that after a single introduction in Spain, CRF47_BF has spread mainly through localized point introductions and subsequent spread in different geographical areas. CRF47_BF is predominant in males (78%) with a predominantly heterosexual transmission (56% of the total, 61% of those with data on transmission mode). The phylodynamic analysis and percent of CRF47 among new HIV diagnoses both support a fluctuating population size of CRF47_BF over the last 15 years with periods of expansion and contraction, suggesting that continued monitoring of this novel variant will be important to track its spread.

It is interesting to note that one cluster of 17 individuals, associated with Navarre, where 14 of 15 individuals with available data were male, was significantly associated (*p* = 0.0388) with transmission among MSM. Although three men were reported to be heterosexual, considering the great male preponderance in the cluster, it is probable that they are non-disclosed MSM (Hué et al., 2014; Ragonnet-Cronin et al., 2018). The identification of an MSM-associated cluster within the CRF47_BF clade may be indicative of the diffusion of CRF47_BF from a heterosexual-driven network to a MSM-driven network. A similar phenomenon has been observed for the two other CRFs of South American origin identified by us in Spain: CRF66_BF (Bacqué et al., 2021) and CRF89_BF (Delgado et al., 2021). Such phenomenon may reflect the migration of these CRFs from countries where heterosexual transmission is predominant to Spain, where most currently expanding HIV-1 clusters are associated with MSM (Patiño-Galindo et al., 2017; Gil et al., 2022). It should be pointed out, however, that outside of the Navarre cluster, the male:female ratio was 3.2:1, which contrasts to the 2.4:1 ratio of self-declared heterosexual men to MSM (decreasing to 1.5:1 if all men with non-specified sexual transmission were MSM). This discrepancy could also be explained by the presence of non-disclosed MSM among self-declared heterosexual men outside of the Navarre cluster, and indicates that epidemiological data on transmission route based on self-reported sexual behaviors should be interpreted with caution.

The recent expansion in Spain of CRF47_BF, whose Brazilian origin is first reported here, is one more example of the increasing relationship of the South American and European HIV-1 epidemics, also reflected in the propagation in Europe of other CRFs (12_BF, 17_BF, 60_BC, 66_BF, and 89_BF) (Simonetti et al., 2014; Fabeni et al., 2015, 2020; Bacqué et al., 2021; Delgado et al., 2021) and variants of subtypes F1 and C (Tovanabutra et al., 2003; de Oliveira et al., 2010; Thomson et al., 2012; Lai et al., 2014; Carvalho et al., 2015; Delgado et al., 2015; Vinken et al., 2019) of South American ancestry, which probably derives from increasing migratory flows from South America to Europe.

The repeated introduction and expansion in Spain of multiple CRFs and non-B subtypes (Delgado et al., 2015, 2019; Patiño-Galindo et al., 2017; González-Domenech et al., 2018; Kostaki et al., 2019) justifies the establishment of a HIV-1 molecular epidemiological surveillance system, aimed at promptly detecting the propagation of such variants, as well as rapidly expanding clusters, that could provide information in real-time on changes in the genetic composition and the dynamics of the HIV-1 epidemic to guide the implementation of preventive public health interventions (Paraskevis et al., 2016; German et al., 2017; Oster et al., 2018). Nevertheless, phylogenetic analyses of HIV sequence data should be taken with caution as recombination can impact phylogenetic inference (Schierup and Hein, 2000; Posada and Crandall, 2002) suggesting network approaches might be better suited for representation of such data (Clement et al., 2000). Nevertheless, we focus on a specific set of CRF47_BF sequences with a shared mosaic structure and therefore our results should be robust to the impacts of recombination (see Figure 3).

## Data Availability Statement

The datasets presented in this study can be found in online repositories. The names of the repository/repositories and accession number(s) can be found below: https://www.ncbi.nlm.nih.gov/genbank/, OK148895-OK148974.

## Ethics Statement

The studies involving human participants were reviewed and approved by the Committee of Research Ethics of Instituto de Salud Carlos III, Majadahonda, Madrid, Spain (report numbers CEI PI 38_2016-v3 and CEI PI 31_2019-v5). Written informed consent for participation was not required for this study, as it used samples and data collected as part of routine clinical practice and patients’ data were anonymized without retaining data allowing individual identification.

## Members of the Spanish Group for the Study of New HIV Diagnoses

Hospital Universitario de Basurto: José Luis Díaz de Tuesta del Arco, Silvia Hernáez, Sofía Ibarra-Ugarte, Josefa Muñoz, María Carmen Nieto-Toboso, and Miren Zuriñe Zubero-Sulibarria. Hospital Universitario de Cruces, Bilbao: Elena Bereciartua-Bastarrica, Luis Elorduy, Ane Josune Goikoetxea-Agirre, and Leyre López-Soria. Hospital Universitario de Galdakao: María José López de Goikoetxea. Hospital Universitario Donostia, San Sebastián: Maitane Aranzamendi, Gustavo Cilla, José Antonio Iribarren, and Yolanda Salicio. Hospital Universitario Araba, Vitoria: Carmen Gómez, and José Joaquín Portu. Hospital Universitario de Navarra, Pamplona: Aitziber Aguinaga, Carmen Ezpeleta, Carmen Martín-Salas, and María Gracia Ruiz-Alda. Hospital Reina Sofía, Tudela: José Javier García-Irure. Hospital Universitario Sant Joan d’Alacant: Fernando Buñuel and Francisco Jover-Díaz. Complejo Hospitalario Universitario de Vigo: Jorge Julio Cabrera, Antonio Ocampo, and Celia Miralles. Complejo Hospitalario Universitario de Pontevedra: Julio Diz-Aren and Matilde Trigo. Complejo Hospitalario Lucus Augusti, Lugo: María José Gude, Ramón Rabuñal, and Eva María Romay. Complejo Hospitalario Universitario de Ourense: Ricardo Fernández-Rodríguez and Juan García-Costa. Hospital Universitario Miguel Servet, Zaragoza: Piedad Arazo and Ana María Martínez-Sapiña. Centro Sanitario Sandoval, Madrid: Jorge del Romero. Hospital Universitario Río Hortega, Valladolid: Belén Lorenzo-Vidal. Hospital Universitario de Toledo: César Gómez.

## Author Contributions

MT, ED, and MP-L conceived of the project. ED collected sequence data from the samples. GH, KC, MP-L, MT, and ED conducted the data analyses. HG performed the data curation. SB, VM, MS, JC-G, and EG-B performed the experimental work. The members of the Spanish Group for the Study of New HIV Diagnoses collected the samples and clinical and epidemiological data for the study. KC, GH, and MP-L wrote the original draft of the manuscript. MT, ED, and HG edited the manuscript. All authors read and approved the manuscript.

## Conflict of Interest

The authors declare that the research was conducted in the absence of any commercial or financial relationships that could be construed as a potential conflict of interest.

## Publisher’s Note

All claims expressed in this article are solely those of the authors and do not necessarily represent those of their affiliated organizations, or those of the publisher, the editors and the reviewers. Any product that may be evaluated in this article, or claim that may be made by its manufacturer, is not guaranteed or endorsed by the publisher.
