# Supplementary material for: The Origin, Epidemiology, and Phylodynamics of Human Immunodeficiency Virus Type 1 CRF47_BF
Source: Front Microbiol. 2022 May 16;13:863123. doi: 10.3389/fmicb.2022.863123 (PMC9172993; doi:10.3389/fmicb.2022.863123)
Supplement: Supplementary file 1 [file Data_Sheet_1.PDF]

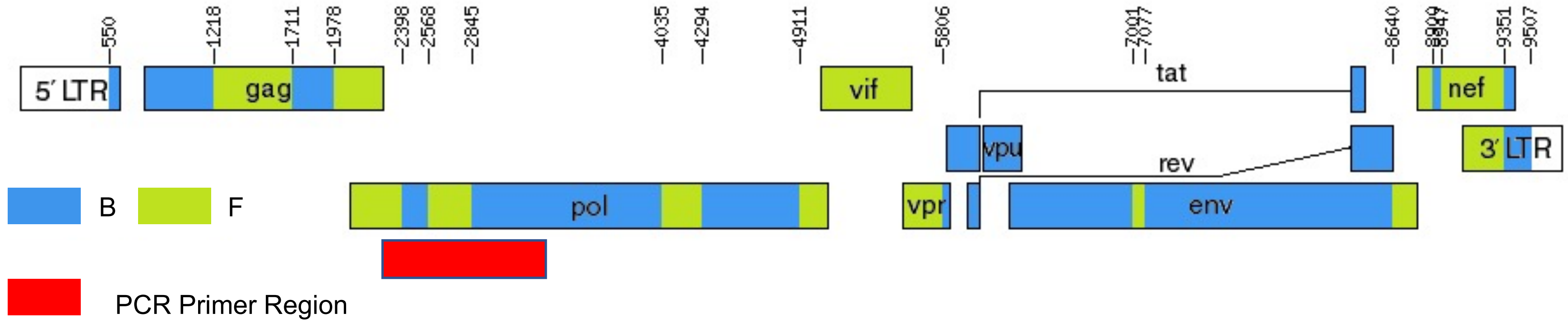

Supplementary Figure 1. Mosaic genomic structure of CRF47_BF (from Los Alamos National Lab HIV Database) with targeted primers for the PR_RT amplicon annotated (shown in red) on the pol gene.
