# Supplementary material for: The Origin, Epidemiology, and Phylodynamics of Human Immunodeficiency Virus Type 1 CRF47_BF
Source: Front Microbiol. 2022 May 16;13:863123. doi: 10.3389/fmicb.2022.863123 (PMC9172993; doi:10.3389/fmicb.2022.863123)
Supplement: Supplementary file 2 [file Data_Sheet_2.PDF]

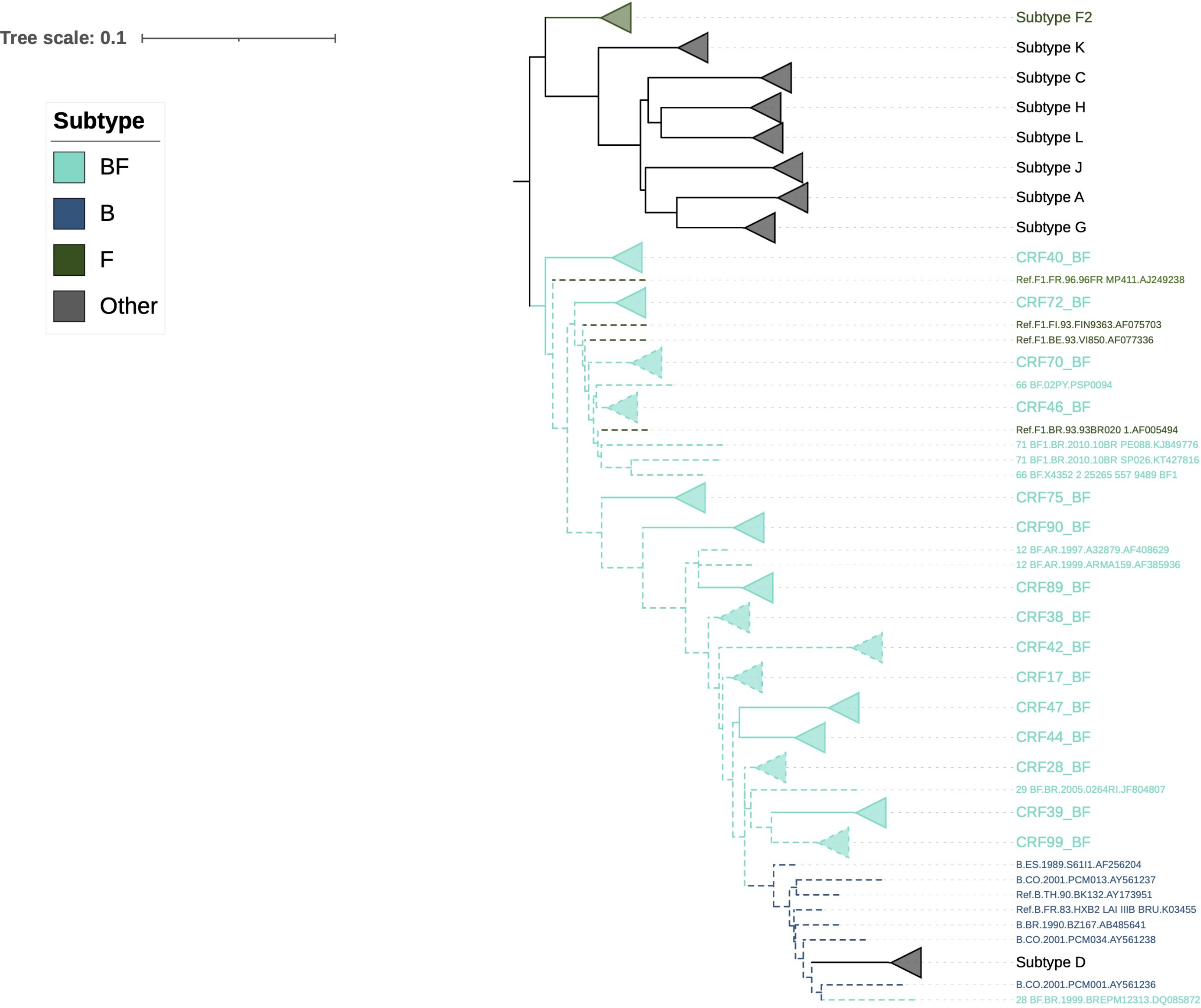

Supplementary Figure 2. Maximum likelihood estimate of phylogenetic relationships amongst the HIV-1 M group subtypes and the CRFx_BFs with a focus on subtypes B and F. Lineages shown with dashed lines have <70% bootstrap support, whereas lineages shown in solid lines have ≥70% bootstrap support. Note phylogenetic relationships among subtypes are impacted by recombination, yet our target group of CRF47_BF sequences form a well supported monophyletic group.
