# Supplementary material for: The Origin, Epidemiology, and Phylodynamics of Human Immunodeficiency Virus Type 1 CRF47_BF
Source: Front Microbiol. 2022 May 16;13:863123. doi: 10.3389/fmicb.2022.863123 (PMC9172993; doi:10.3389/fmicb.2022.863123)
Supplement: Supplementary file 3 [file Data_Sheet_3.PDF]

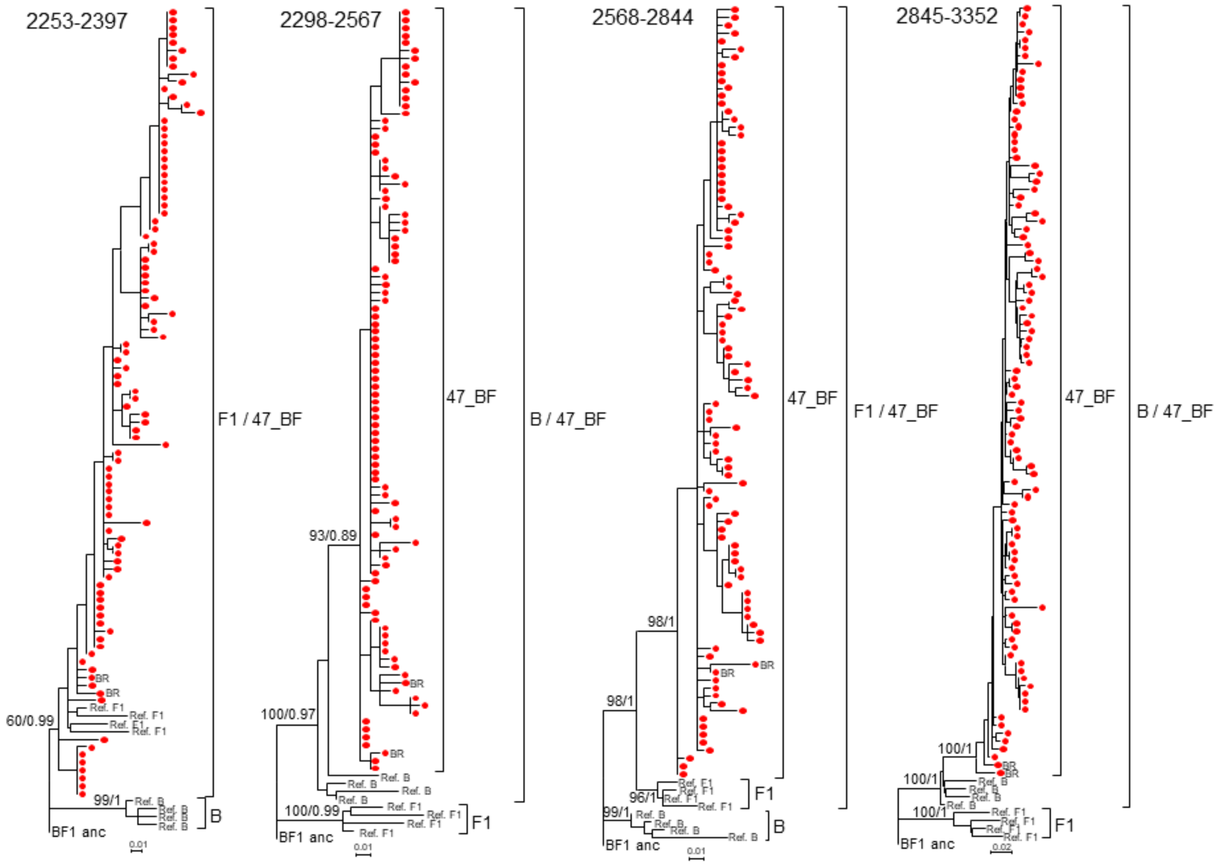

Supplementary Figure 3. Phylogenetic trees of PR-RT segments of CRF47_BF sequences. HXB2 positions delimiting segments are shown on the upper left side of each tree, and correspond to previously defined CRF47 breakpoints (Fernández-García et al., 2010). CRF47_BF sequences are labeled with red circles. Those from Brazil additionally have the BR label. B and F1 subtype references are labeled with Ref. Trees are rooted with a reconstructed B-F1 ancestor sequence (BF1 anc) to avoid the long-branch attraction artifact that may occur when using outgroups too distant from the ingroup, which may cause collapse of ingroup subclades, similarly to the approach used in other studies (Thomson and Fernández-García, 2011; Travers et al., 2004; Seager et al., 2014). Trees were constructed via maximum likelihood with two programs: W-IQ-Tree (Trifinopoulos et al., 2016), using the best-fit evolutionary model selected by the program and ultrafast bootstrap node support values; and PhyML (Guindon et al., 2010), using the best-fit evolutionary model selected by SMS program (Lefort et al., 2017) and aLRT SH-like node support values. Support values of nodes corresponding to B, F1, CRF47, B/CRF47, and F1/CRF47 clades are indicated, in this order, with ultrafast bootstrap/aLRT SH-like values.

References

Fernández-García, A., Pérez-Álvarez, L., Cuevas, M. T., Delgado, E., Muñoz-Nieto, M., Cilla, G., et al. (2010). Identification of a new HIV type 1 circulating BF intersubtype recombinant form (CRF47_BF) in Spain. *AIDS Res. Hum. Retroviruses* 26, 827–832.

Guindon, S., Dufayard, J. F., Lefort, V., Anisimova, M., Hordijk, W., and Gascuel O. (2010). New algorithms and methods to estimate maximum-likelihood phylogenies: assessing the performance of PhyML 3.0. *Syst. Biol.* 59, 307-321. doi:10.1093/sysbio/syq010.

Lefort, V., Longueville, J. E., Gascuel, O (2017). SMS: Smart Model Selection in PhyML. *Mol. Biol. Evol.* 34, 2422-2424. doi: 10.1093/molbev/msx149.

Seager, I., Travers, S. A., Leeson, M. D., Crampin, A. C., French, N., Glynn, J. R., et al. (2014). Coreceptor usage, diversity, and divergence in drug-naive and drug-exposed individuals from Malawi, infected with HIV-1 subtype C for more than 20 years. *AIDS Res. Hum. Retroviruses* 30, 975–983. doi: 10.1089/aid.2013.0240.

Thomson, M. M., and Fernández-García, A. (2011). Phylogenetic structure in African HIV-1 subtype C revealed by selective sequential pruning. *Virology* 415, 30–38. doi: 10.1016/j.virol.2011.03.021.

Travers, S. A., Clewley, J. P., Glynn, J. R., Fine, P. E., Crampin, A. C., Sibande, F., et al. (2004). Timing and reconstruction of the most recent common ancestor of the subtype C clade of human immunodeficiency virus type 1. *J. Virol.* 78, 10501–10506. doi: 10.1128/JVI.78.19.10501-10506.2004.

Trifinopoulos, J., Nguyen, L. T., von Haeseler, A., and Minh, B. Q. (2016). W-IQ-TREE: a fast online phylogenetic tool for maximum likelihood analysis. *Nucleic Acids Res.* 44, W232–W235. doi: 10.1093/nar/gkw256.
